# Acoel regeneration mechanisms indicate an ancient role for muscle in regenerative patterning

**DOI:** 10.1038/s41467-017-01148-5

**Published:** 2017-10-30

**Authors:** Amelie A. Raz, Mansi Srivastava, Ranja Salvamoser, Peter W. Reddien

**Affiliations:** 1 Howard Hughes Medical Institute, MIT Biology, Whitehead Institute for Biomedical Research, 9 Cambridge Center, Cambridge, MA 02142 USA; 2 Department of Organismic and Evolutionary Biology, Harvard University, Cambridge, MA 02138 USA; 3 Present Address: Walter and Eliza Hall Institute of Medical Research, Parkville, VIC 3052 Australia

## Abstract

Positional information is required for animal regeneration, yet how it is harbored in adult tissues is poorly understood. In planarians, positional control genes (PCGs) control regeneration outcomes and are regionally expressed predominately in the musculature. Acoels are early diverging bilaterally symmetric animals, having separated from other bilaterians > 550 million years ago. Here, we find that PCGs in the acoel *Hofstenia miamia* are expressed together and specifically in a primary differentiated cell type: muscle. The vast majority of *Hofstenia* muscle cells in regions tested express PCGs, suggesting positional information is a major feature of muscle. PCG expression domains are dynamic in muscle after injury, consistent with known PCG roles in guiding regeneration. These data demonstrate an instructive positional role for *Hofstenia* muscle and this similarity with planarians suggests mesodermal muscle originated at the base of the Bilateria not only for contraction, but also as the source of positional information guiding regeneration.

## Abstract (web summary)

Planarian regenerative mechanisms suggest that muscle has an instructive role in patterning. Here, the authors show that muscle is also responsible for regenerative patterning information in an early-branching bilaterian, *Hofstenia miamia*, dating this back to the dawn of the Bilateria, over 550 million years ago.

## Introduction

The ability to regenerate is widespread in the animal kingdom^1^. However, whether similar or distinct underlying mechanisms explain regeneration across diverse animals remains unclear. Positional information refers to the molecular processes by which the regional identity of cells and tissues can be specified^2^. Positional information is necessary for regulating regeneration, yet the identity of adult cell types across animals that are the source of positional information involved in regeneration is poorly understood^3, 4^. In one classical model system for studying regeneration, planarian flatworms, muscle cells have been identified as the primary site of expression of patterning genes^5^. This specific expression of patterning molecules indicates that muscle is a major source of flatworm adult positional information^5^. Muscle was subsequently found to specifically express regionally restricted Wnts in the regenerative flatworm parasite *Echinococcus multilocularis* ^6^. Mesoderm, and specifically mesoderm-derived muscle, arose at the base of the Bilateria over 550 million years ago^7–13^. We sought to test whether muscle originated in evolution not only for generating contractile force, but also as the tissue responsible for the control of regenerative patterning. This would suggest a broad role for muscle in controlling regeneration across bilaterians.

**Fig. 1:**
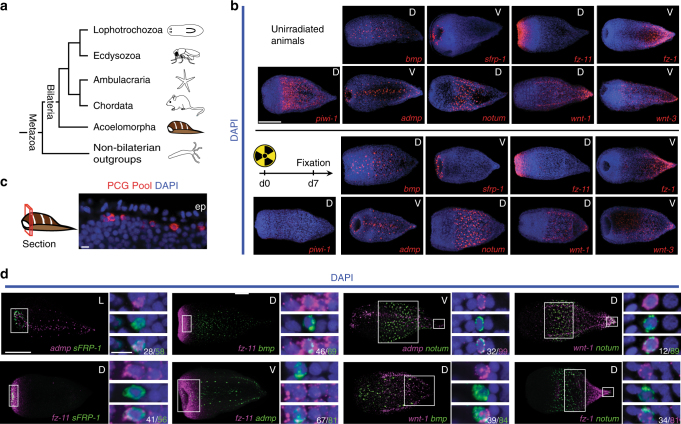
PCGs are expressed in a common differentiated cell type. **a** A phylogenetic tree representing the relative position of acoels within the Metazoa. Phylogenetic analyses support the position of acoels as the sister group to other bilaterian lineages, diverging shortly after the origin of the Bilateria > 550 million years ago^17, 18, 20^. **b** As a marker for cycling cells, *piwi-1* expression is sensitive to irradiation and is ablated by 7 days post-irradiation with 10,000 rads^17^. PCG expression domains^17^ were maintained, suggesting that they are primarily expressed in non-cycling, differentiated cells. **c** Fluorescent image of a transverse optical section in the anterior. ep epidermis. PCG pool comprised of simultaneously hybridized *sFRP-1, frizzled-11, bmp, admp, notum, wnt-1*, and *frizzled-1* RNA probes. **d** Eighteen RNA probe pairs were used in double-FISH experiments. The denominator for each quantification is the total number of cells in the channel indicated (i.e., magenta or green); the numerator is the total number of double-positive cells for that pair. Quantification was performed in area indicated by the white box, and inset panels below are higher magnification images of cells for this region. See Supplementary Fig. 1b for remaining pairs. Scale bars: **b** 100 μm; **c** 10 μm; **d** 100 μm, overview image; 10 μm, cell inset. D dorsal view, V ventral view, L lateral view. Overview images are representative of 5/5 animals examined

Acoels, an enigmatic taxon of marine worms, are highly regenerative and have recently been highlighted as a model for understanding the evolution of regeneration mechanisms^14–17^. Recent phylogenetic work using transcriptome-level sequences supports a position for the acoels, together with nemertodermatids and *Xenoturbella*, as a sister clade to all other bilaterians (the bilaterally symmetric animals) (Fig. 1a)^17–22^. This phylogenetic placement of acoels suggests that they diverged from other bilaterians over 550 million years ago^7^. This position is significant for experimental approaches to animal biology because comparison of biology in acoels, serving as an outgroup to other bilaterians, can point to attributes likely present in the last common ancestor of the Bilateria. By assessing the role of muscle in regenerative patterning in an acoel, we can seek to determine the ancestral and broadly conserved functions of muscle.

*Hofstenia miamia* is an acoel capable of whole body regeneration^17, 23–25^. We recently developed *Hofstenia* as a new and powerful model for studying regeneration^17^. *Hofstenia* express molecules constitutively in adult tissues that are required for regional tissue identity in regeneration^17^. Similar genes in planarians have been referred to as positional control genes (PCGs), which are genes that display constitutive regionalized expression and either a patterning abnormal RNAi phenotype or encode a protein predicted to act in the pathway of such a patterning molecule^5^. In *Hofstenia*, genes encoding several Wnt pathway components, including Wnt signaling ligands, Frizzled receptors, and secreted antagonists (e.g., secreted frizzled-related protein, sFRP, or the secreted Wnt deacylase Notum^26, 27^) are regionally expressed along the *Hofstenia* anterior–posterior (AP) axis^17^. Perturbation of several Wnt pathway components resulted in abnormal AP patterning in regeneration^17^. For instance, RNAi of the posteriorly expressed *wnt-1* gene resulted in regeneration of a head in place of a tail, generating two-headed acoels, whereas RNAi of the Wnt-antagonist *notum* resulted in two-tailed acoels. Genes encoding several Bmp pathway components, including Bmp-family signaling ligands, are expressed in different patterns on the dorsal–ventral (DV) and medial–lateral (ML) axes^17^. RNAi of the dorsally expressed *bmp* gene or the ventrally expressed *admp* gene resulted in abnormal dorsal–ventral regenerative patterning^17^. These Wnt and Bmp signaling pathways are, therefore, hypothesized to instruct the regeneration of correct axial structures after amputation and involve regional and constitutive expression of signaling ligands, receptors, and secreted pathway inhibitors^17^.

Here, we show that *Hofstenia* PCGs are co-expressed with one another in a common differentiated, subepidermal cell type, consistent with a single primary source of adult positional information. Analysis by *in situ* hybridization and single-cell quantitative real-time PCR (qRT-PCR) demonstrated that all known *Hofstenia* PCGs are specifically expressed in muscle cells during both homeostatic tissue maintenance and regeneration. In tested regions, *Hofstenia* muscle cells expressed one or more PCGs, suggesting that most *Hofstenia* muscle cells express patterning molecules as well as control body contraction. The expression domains of PCGs shifted in muscle after amputation, consistent with muscle guiding regenerative patterning choices. These data demonstrate that despite being separated by over 550 million years of evolution, acoels, like planarians, use muscle as a physical positional map to guide tissue turnover and regenerative patterning. This is consistent with a model in which mesodermal muscle was the primary source of regenerative positional information in the last common ancestor of the Bilateria and has broad instructive roles in regeneration in extant animals.

## Results

### *Hofstenia* PCGs are expressed in a common differentiated cell type

We found that all *Hofstenia* PCGs examined were primarily expressed in a peripheral, subepidermal cell layer of non-cycling differentiated cells (Fig. 1b, c, Supplementary Fig. 1a, Supplementary Table 2). Eighteen *Hofstenia* PCG pairs with overlapping expression domains were examined for co-expression by double fluorescence *in situ* hybridization (FISH) (Fig. 1d and Supplementary Fig. 1b). In most instances where the expression domains of PCG pairs overlapped, the tested genes were expressed together in the same cells to a substantial degree (Fig. 1d and Supplementary Fig. 1b). This is consistent with the possibility that a primary single cell type, such as muscle, might harbor *Hofstenia* positional information.

### *Hofstenia* muscle produces positional information

**Fig. 2:**
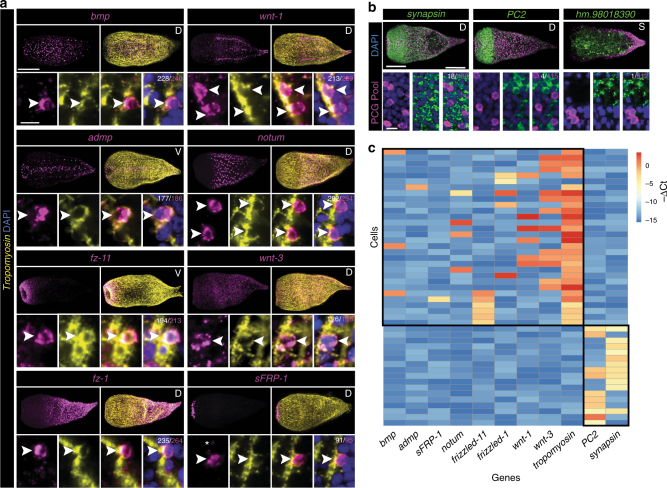
Muscle specifically harbors positional information in *Hofstenia*. **a** PCG( + ) cells also expressed *tropomyosin*. Both muscle fibers and nuclei are marked by *tropomyosin* mRNA; PCG mRNA is localized primarily around the nucleus. Denominator for each quantification is the total number of PCG-positive cells; the numerator is the total number of PCG/*tropomyosin* double-positive cells. Asterisk denotes fiber expression of PCG. **b** PCGs were not significantly expressed in *PC2*- or *synapsin*-positive neuron populations, nor in an internal differentiated cell population marked by expression of *hm.98018390*. The PCG RNA probe pool was the same as in Fig. 1c. **c** Single-cell cDNA libraries were screened by qRT-PCR with primers specific to PCGs, tissue-type markers (*tropomyosin, PC2*, and *synapsin*), and a housekeeping gene *GAPDH*. Any cell without expression of *GAPDH* was excluded from the analysis. Shown are all remaining cells positive for at least one PCG and/or positive for at least one neural marker. −ΔCt = −[Ct(gene of interest)−Ct(*GAPDH*)]. All Ct values greater than 32 or undetected were analyzed as Ct = 32. 29/30 PCG-positive cells also expressed *tropomyosin*. 0/30 PCG-positive cells expressed the neural markers *PC2* or *synapsin*. *tropomyosin* expression was never found in single-cell libraries with expression of either neural marker, indicating that our libraries were indeed amplified from single cells. Scale bars: **a**, **b** 100 μm, overview image; 10 μm, cell inset. D dorsal view, V ventral view, L lateral view, S coronal section. Overview images are representative of 5/5 animals examined

To test whether *Hofstenia* PCG expression was in muscle, we developed a FISH protocol that allowed detection of the nuclei connected to muscle fibers, involving visualizing *tropomyosin* messenger RNA (mRNA) transcripts within individual cells (Fig. 2a and Supplementary Fig. 2a–c). *Hofstenia* muscle cells are organized into long fibers; dissociation of muscle fibers reveals that individual muscle cells appear to be mononucleate (Supplementary Fig. 2d). These muscle fibers are comprised of several subtypes, including body-wall longitudinal and circular fibers, as well as fibers that radiate from or encircle the pharynx; all of these subtypes are marked by *tropomyosin* expression^17, 28^ (Supplementary Fig. 2a).

Strikingly, every PCG analyzed was co-expressed with *tropomyosin* to a high degree (89%–96% of PCG-positive cells also expressed *tropomyosin*) (Fig. 2a). PCG transcripts were predominantly perinuclearly localized in these cells. Because PCGs largely encode secreted or transmembrane proteins, this localization likely reflects perinuclear association with ribosomes on the endoplasmic reticulum. We could not confidently identify every muscle nucleus with this method because of relatively weak perinuclear *tropomyosin( + )* signal with FISH, indicating our quantification of the percentage of PCG+ cells that expressed *tropomyosin* was an underestimate of the degree of overlap, and PCG expression may be almost exclusive to muscle cells.

We also identified an additional *Hofstenia* muscle marker, *titin-like*, with mRNA localized primarily along muscle fibers (Supplementary Fig. 2e). Co-expression analyses with this alternative marker also showed restriction of PCG expression to muscle (Supplementary Fig. 2e). The PCG pairs that were not robustly co-expressed together (e.g., *bmp* and *notum*) were nonetheless individually each expressed in *tropomyosin*(* + *) cells, indicating that some PCGs are expressed in different subsets of muscle cells (Supplementary Fig. 1b, Fig. 2a). We conclude that a major site of PCG expression in *Hofstenia* is muscle.

To test the specificity of PCG expression to muscle cells, we assessed by FISH whether there was any co-expression of PCGs with other cell types. PCGs were not significantly expressed in neurons (*synapsin + *or *PC2 + *cells) or an internal differentiated cell type marked by *hm.98018390* expression (Fig. 2b). The cellular source of regeneration in *Hofstenia* is a cycling, mesenchymal *piwi-1* + cell population^17^. PCG expression was primarily non-overlapping with *piwi-1 + *cells (Supplementary Fig. 2f). One PCG, *frizzled-1*, was expressed in some *piwi-1* + cells, as well as in *piwi-1(−)* cells (Supplementary Fig. 2f). In principle, *frizzled-1* could encode a Wnt receptor that participates in both Wnt signaling within differentiated cells and in transducing signals from differentiated cells to responsive *piwi-1( + )* cycling cells.

We next utilized single-cell qRT-PCR to assess the identity of cells expressing PCGs (Fig. 2c). This approach allowed quantification of the fraction of PCG + cells that were positive for muscle markers and further assessment of the specificity of PCG expression in muscle. Animals were dissociated and cells were individually sorted into wells of a 96-well plate using fluorescence activated cell sorting (FACS) (Supplementary Fig. 2g). Complementary DNA (cDNA) libraries were generated from the single cells and assayed by qRT-PCR for expression of PCGs, *tropomyosin*, and the neural markers *PC2* and *synapsin*. The overwhelming majority (29/30) of PCG + single cells were muscle (*tropomyosin* + ) (Fig. 2c). Furthermore, no PCG-positive cell (0/30) expressed either neural marker (Fig. 2c).

**Fig. 3:**
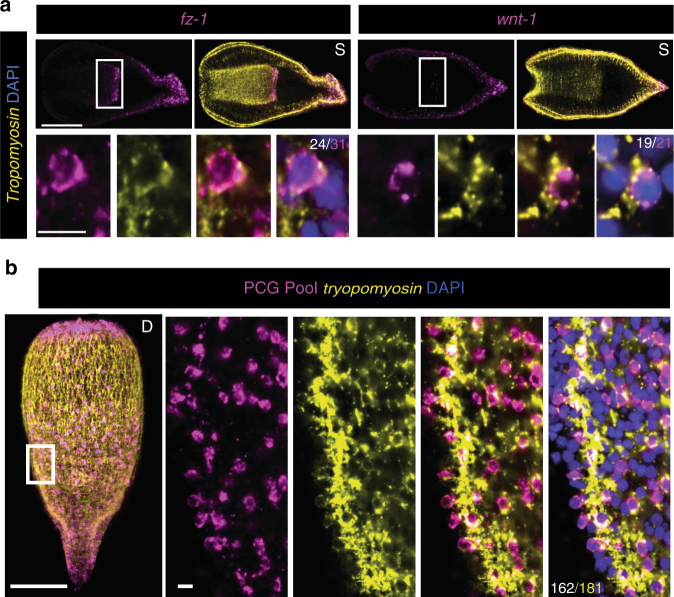
PCG expression is a major attribute of *Hofstenia* muscle. **a** The posterior PCGs *frizzled-1* and *wnt-1* were expressed both in the body wall and interiorly. The interior expression was specific to *tropomyosin*-positive pharyngeal muscle fibers. **b** Co-expression of PCGs and *tropomyosin*, detected with hybridization of seven pooled PCG probes as in Figs. 1c and 2b. Quantification was specific for region shown. Denominator is total number of *tropomyosin*-positive nuclei; numerator is the total number of double-positive. **a**, **b** 100 μm, overview image; 10 μm, cell inset. D dorsal view, S coronal section. Overview images are representative of 5/5 animals examined

Although most PCGs were specifically expressed in cells near the body wall, some were expressed internally, including *wnt-1* and *frizzled-1*. The internal expression of both of these PCGs was specifically in pharyngeal muscle cells (Fig. 3a). Altogether, these data suggest that muscle is the major tissue responsible for expression of all known patterning genes in adult *Hofstenia*.

To determine the degree to which muscle cells express one or more PCGs, RNA probes against seven different PCGs were pooled and simultaneously utilized for hybridization with endogenous transcripts (Fig. 3b). In a central body region analyzed, where multiple known PCG expression zones exist, the vast majority of muscle cells identified (89%) also expressed at least one PCG (Fig. 3b). This observation suggests that, in addition to contraction, a major function of *Hofstenia* muscle cells is maintenance of adult tissue positional identity (Fig. 3b).

### Muscle cells dynamically re-express PCGs during regeneration

**Fig. 4:**
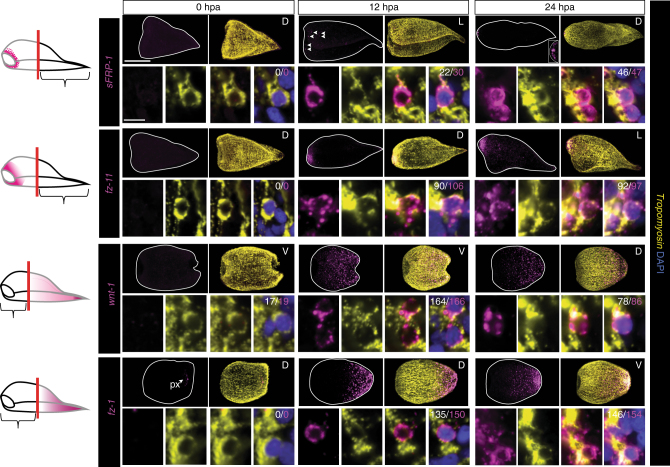
PCGs are re-expressed in muscle during regeneration. Animals were amputated to remove expression zones of anteriorly or posteriorly restricted genes. In the schematic, the black outline represents the regenerating piece assayed; grey outline represents the piece removed. Successful removal of body wall expression region was verified by observation of PCG expression at the time of amputation (0 h post-amputation [hpa]). The fraction of PCG-positive cells that expressed *tropomyosin* was assayed at 12 and 24 hpa. Intact interior pharynx (px) expression was excluded from analysis. The denominator for each quantification is the total number of PCG-positive cells; the numerator is the total number of PCG/*tropomyosin* double-positive cells. 100 μm, overview image; 10 μm, cell inset. D dorsal view, V ventral view, L lateral view. Overview images are representative of 5/5 animals examined

If muscle-specific PCG expression is responsible for patterning new tissues during regeneration, PCGs must be rapidly re-expressed in muscle cells after amputation of their expression zones. To test this prediction, we assessed re-expression of PCGs with restricted expression along the AP axis following amputation. Some PCGs were significantly re-expressed as early as 12 h post-amputation (hpa), and all PCGs examined were re-expressed in domains reflecting their normal axial regions by 24 hpa (Fig. 4). At both of these time points, the vast majority of these newly PCG-positive cells were identifiably muscle cells, consistent with the hypothesis that muscle expression of PCGs is responsible for early patterning decisions in regeneration (Fig. 4).

Single-cell qRT-PCR was also performed on cells isolated from 24 hpa regenerating *Hofstenia* (Supplementary Fig. 3a). Eight out of ten PCG( + ) cells coexpressed *tropomyosin* by this assay, and none expressed the neural markers *PC2* or *synapsin*. The remaining two *tropomyosin*(−), PCG( + ) cells expressed the cycling cell marker *piwi-1*. These two cells could represent a muscle-progenitor class that is enriched during regeneration. Similar PCG expression in rare cycling neoblast cells can be observed in planarians^29^.

**Fig. 5:**
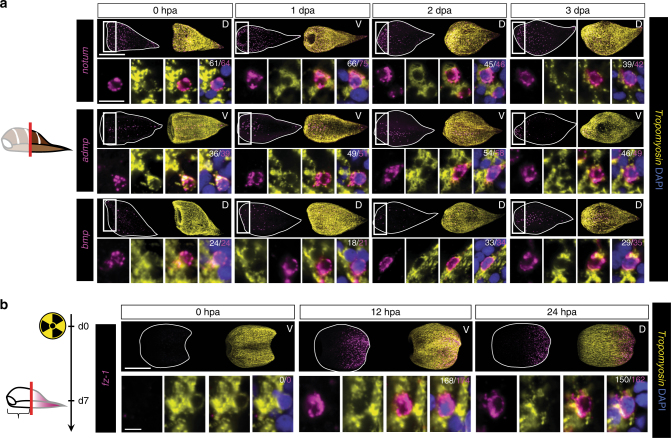
Muscle cells dynamically re-express PCGs during regeneration. **a** Animals underwent decapitation and the fraction of PCG-positive cells that expressed *tropomyosin* was assayed during blastema formation at 0, 1, 2, and 3 days post-amputation (dpa). The denominator for each quantification is the total number of PCG-positive cells; the numerator is the total number of PCG/*tropomyosin* double-positive cells. Quantification was performed in area indicated by the white box, and inset panels below are higher magnification images of cells for this region. **b** Animals were lethally irradiated with 10,000 rads and amputated after ablation of *piwi-1* expression (7 days post-irradiation). PCG expression was assayed 12 and 24 hpa. The denominator for each quantification is the total number of PCG-positive cells; the numerator is the total number of PCG/*tropomyosin* double-positive cells. Animals were from the same cohort as Supplementary Fig. 3b, c. Scale bars: **a**, **b** 100 μm, overview image; 10 μm, cell inset. D dorsal view, V ventral view. Overview images are representative of 5/5 animals examined

Following this early phase of regeneration (0–24 hpa), amputated *Hofstenia* form regenerative outgrowths, termed blastemas, at wound sites^17^. New tissues are formed and patterned in these blastemas. We assessed several PCGs with broader expression across the AP axis to determine if there is a transient state of non-muscle PCG expression during blastema formation. PCG( + ) cells at the wound site or in the blastemas at 1–3 days post-amputation (dpa) were almost exclusively muscle cells (Fig. 5a), suggesting that muscle acts as the major patterning tissue throughout all stages of *Hofstenia* tissue maintenance and regeneration.

The rapid re-expression of PCGs after amputation could involve production of new muscle cells with a specified positional program, and/or re-expression of PCGs in existing muscle cells, which would suggest that muscle cells are capable of dynamic changes in their positional identity. To explore the latter possibility, we used irradiation (10,000 rads) to ablate all cycling cells^17^ (Supplementary Fig. 3b). Post-irradiation, animals were amputated to remove specific PCG expression zones, and then assayed at 12 and 24 hpa for re-expression of those PCGs. Several PCGs assayed were not re-expressed after amputation in this assay, suggesting that re-expression of some PCGs requires new tissue production from cycling cells, or some other influence of the cycling cells. (Supplementary Fig. 3c). However, the posteriorly expressed PCG *frizzled-1* clearly displayed irradiation-insensitive re-expression within muscle of amputated head fragments, consistent with the possibility that existing muscle cells are capable of dynamically changing their gene expression program to reflect their new positional identity after amputation (Fig. 5b). This is consistent with a role for muscle in guiding patterning outcomes during regeneration.

## Discussion

**Fig. 6:**
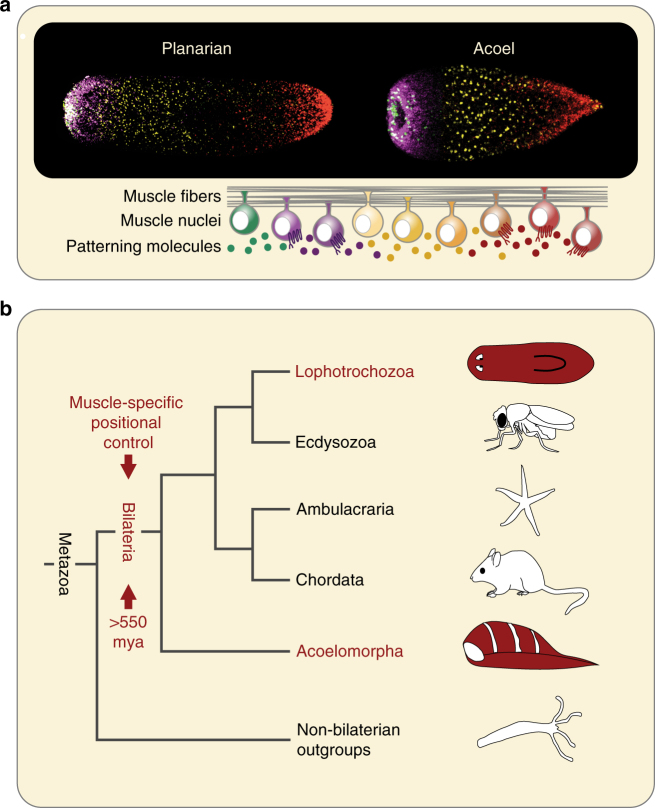
Model for evolution of muscle-specific positional control. **a** Both planarians and *Hofstenia* demonstrate muscle-specific expression of positional control genes, despite divergence of over 550 million years. **b** Muscle-specific expression of PCGs in both lophotrochozoans and acoels suggests that it arose as a mechanism of regenerative patterning at the base of the Bilateria, over 550 million years ago

The phylogenetic position of *Hofstenia* as part of a sister clade to other bilaterians allows for a powerful point of comparison with other organisms to identify processes likely present in the last ancestor of all bilaterians (Fig. 6a, b)^14^. Such processes are potential candidates to be broadly important in the biology of extant bilaterians. The development of mesoderm, and of mesodermal muscle, is a major evolutionary innovation that appeared in and is specific to the bilaterians^8–13^. Mesodermal muscle might be one of the earliest mesodermal tissue innovations^30, 31^. Our data demonstrating an instructive positional role for muscle in *Hofstenia* raises the possibility that muscle originated at the base of the Bilateria not only as a contractile tissue, but also for expressing a physical positional map responsible for guiding regenerative patterning (Fig. 6a, b). Although independent evolution of muscle-driven regenerative patterning in acoels and flatworms is possible, the similarities in the details of this system are more parsimonious with a model of homology. The individual genes and the pathways active in regenerative patterning of acoels and flatworms are similar, exhibit similar expression patterns and functions, and the tissue responsible for their production is also the same. This model can be further investigated in diverse other clades. Our findings demonstrate the use of muscle in regenerative patterning in organisms separated by > 550 million years of evolution, and predict that regenerative positional information might be widely active in muscle, or other similar mesodermal derivatives, in diverse extant bilaterians.

## Methods

### Cloning

*titin-like* (forward primer 5′GTTGGCTTGGCGGAAGTA-3′*;* reverse primer 5-′ATCTCAAGCGGGACCACA-3′) and *ho.98018390* (forward primer 5′CGCTGTTGTCCAGACGATT-3′; reverse primer 5′TCTTGGTCTGCCCCAACT3′) were cloned from cDNA into the pGEM vector (Promega). *titin-like* was annotated by best BLAST hit.

### Fixation and *in situ* Hybridizations

Animals were relaxed in 1% MgCl_2_ in seawater, followed by fixation in 4% paraformaldehyde in 1% phosphate-buffered saline with 0.1% Triton-X (PBST) for one hour, and stored in methanol at −20 °C for up to several months. Digoxigenin (DIG)-, fluorescein isothiocynate (FITC)-, and dinitrophenol (DNP)-labeled riboprobes were synthesized^32^. Animals were treated with 2 μg/mL proteinase K in PBST with 0.1% SDS for 10 min, then with 4% formaldehyde in PBST for 10 min. Animals were rinsed in PBST twice, and washed for 10 min in 1:1 PBST:PreHybe (PreHybe prepared as described^17^), then incubated in PreHybe for two hours at 56 °C, then hybridized with RNA probes diluted 1:800 in Hybe (Hybe prepared as described^17^) for at least 16 h at 56 °C. Animals were blocked for 1 h prior to incubating overnight at 4 °C with anti-DIG-POD (1:1500, Roche), anti-FITC-POD (1:2000, Roche), or anti-DNP-HRP (1:150, Perkin-Elmer) in blocking solutions of PBST with 10% western blocking reagent (Roche) for anti-DIG-POD or with 5% western blocking reagent (Roche) and 5% casein solution (Sigma) for both anti-FITC-POD and anti-DNP-HRP. Fluorescent tyramide development and amplification was performed^33, 34^ by first placing the animals for 5 min in borate buffer (0.1 M boric acid, 2 M NaCl, pH 8.5), followed by 10 min in borate buffer with rhodamine (1:1000) or fluorescein (1:1500) tyramide, and 0.0003% hydrogen peroxide. After development, peroxidase activation was performed in a 1% sodium azide solution for at least 1 h, followed by antibody labeling for the second probe. Animals were stained in 1 μg/mL DAPI (Sigma).

### Maceration for cell *in situ* hybridization

Animals were gently macerated in 45 μL calcium- and magnesium-free artificial sea water (CMF-ASW: 449 mM NaCl, 9 mM KCl, 33 mM Na_2_SO_4_, 2.14 mM NaHCO_3_ 10 mM Tris-Cl, 2.5 mM EGTA) and 5 μL TH Liberase (Roche) at 35° for 2 h. They were diluted with 100 μL CMF-ASW and 15 uL of the cell suspension was added to a poly-d-Lysine coverslip (BioCoat), fixed with 4% PFA in CMF-ASW, and then incubated in PreHybe. Staining protocol was the same as in whole animals.

### Image acquisition and quantification

Fluorescence image acquisition performed using a Zeiss LSM 700 confocal microscope. Image J software (Fiji) was used for processing and quantifying images. Images were quantified manually by first counting the number of (denominator)-positive cells while blinded to the other channel, then by counting the number of second-channel positive cells while blinded to the first channel. These were then overlayed to determine co-expression. See Supplementary Fig. 2c.

### Irradiation

Animals were irradiated using a dual Gammacell-40 ^137^cesium source to deliver 10,000 rads.

### Fluorescence-activated cell sorting

Animals were macerated in calcium- and magnesium-free artificial sea water (CMF-ASW: 449 mM NaCl, 9 mM KCl, 33 mM Na_2_SO_4_, 2.14 mM NaHCO_3_ 10 mM Tris-Cl, 2.5 mM EGTA) with 1% heat-inactivated horse serum by vigorously pipetting for 1–2 min. Cell suspensions were passed through a 40 μm cell strainer (Falcon) and pelleted at 300 g for 5 min at 4 °C, and resuspended in CMF-ASW. After resuspension, cells were once again passed through a 40 um strainer. In all, 10 mg/mL Hoechst solution (Invitrogen) was added to the cell suspension at a 1:400 dilution and incubated in the dark for 45 min. In all, 5 mg/mL propidium iodide was added to allow for detection of permeabilized cells. Cells were stored on ice for no longer than 30 min after addition of propidium iodide before sorting. Cells were sorted on a MoFlo (Beckman-Coulter) with gating inclusive of all Hoechst-positive, propidium iodide-negative events to exclude dead cells and cellular debris. Cells were sorted into 96-well plates pre-loaded with 5 uL Buffer TCL (Qiagen) with 1% 2-mercaptoethanol. Plates were immediately stored at −80 °C.

### Single-cell cDNA library construction

Libraries were prepared using the SmartSeq2 method^35, 36^. In all, 11 uL of Ampure XP beads (Agencourt) were added to each well of a 96-well plate, incubated for 10 min, and put on a 96-well magnet plate (Dynamag 96-side magnet; Life Technologies) for 5 min. Supernatant was removed and beads were washed twice with 80% ethanol. Ethanol was removed and beads were eluted in a mix of 1 uL reverse-transcription primer (5′-AAGCAGTGGTATCAACGCAGAGTACT(30)VN-3′, IDT DNA), 1 uL of dNTP mix (10 mM), 0.1 uL of SUPERase RNase-inhibitor (40 U/uL; Life Technologies), and 1.9 uL of H_2_O. The plate was incubated at 72 °C for 3 min and placed immediately on ice. A mix of 1.65 uL H_2_O, 2 uL of 5× Maxima reverse-transcription buffer (Thermo-Fischer), 0.9 uL MgCl_2_ (100 mM Sigma-Aldrich; M1028), 2 uL of Betaine (5 M; Sigma-Aldrich; B0300-5VL), 0.25 uL of SUPERase RNase-inhibitor (40 U/uL), 0.1 uL of Maxima RNase H- RT (200 U/μL; Thermo-Fischer, EP0753), and 0.1 template switching-oligo (Exiqon; 100 uM; AAGCAGTGGTATCAACGCAGAGTACrGrG + G; r and “ + ’’ denote RNA and LNA bases, respectively) was added to each well. Plate was incubated at 42 °C for 90 min, followed by 10 cycles of (50 °C for 2 min, 42 °C for 2 min), followed by 70 °C for 15 min. Following reverse-transcription a pre-amplification mix of 14 uL was added to each well [1 uL of H_2_O; 0.5 uL of PCR primer (10 uM; 5′-AAGCAGTGGTATCAACGCAGAGT-3′), and 12.5 uL of KAPA HiFi HotStart ReadyMix (Kapa Biosystems; KK2601)]. The cDNA was amplified using the following program: 98 °C for 3 min; 20 cycles of (98 °C for 15 s, 67 °C for 20 s, 72 °C for 6 min); 72 °C for 5 min; hold at 4 °C. Following pre-amplification PCR products were purified using × 0.8 Ampure XP beads, and eluted in 20 uL of H_2_O. Amplified cDNA concentrations were measured using Qubit HS-DNA reagents (Life Technologies). All samples were diluted and normalized to 0.2 ng/uL.

### Quantitative real-time PCR

All qRT-PCR primers were tested to ensure efficiency between 90%–110% and a standard curve *R* ^2^ > 0.98. A 20 uL mix was made in MicroAmp Fast Optical 96-Well Reaction Plate (Applied Biosystems) of 50% EvaGreen (BioRad), 5% forward primer, 5% backwards primer, 2% cDNA, and 38% H_2_O for each single-cell cDNA library. All libraries were initially screened for expression of GAPDH (forward: 5′-TTCCGTGTACCAACACCAGA-3′, backward 5′-GGAGATTGACTGGCATCCTT-3′). Any library without expression of GAPDH (~ 20% of libraries) was excluded from further analysis. Expression was then analyzed for genes of interest. Primers used for qRT-PCR are listed in Supplementary Table 1. All qRT-PCR was performed on a 7500 Fast Real-Time PCR System machine (Applied Biosystems) and analyzed on 7500 Software v2.0.5. All Ct values > 32 or undetected were thresholded to Ct = 32. The reported measure -ΔCt equals -[Ct(gene of interest)−Ct(GAPDH)].

### Data availability

The authors declare that all data supporting the findings of this study are available within the article and its supplementary information files or from the corresponding author upon reasonable request. Nucleotide sequences for *hm.98018390* and *titin-like* have been deposited in the GenBank under accession codes: MF568700 (*hm.98018390*) and MF568701 (*titin-like*).

## Electronic supplementary material

Supplementary Information

Peer Review File
