# Supplementary material for: Acoel regeneration mechanisms indicate an ancient role for muscle in regenerative patterning
Source: Nat Commun. 2017 Oct 30;8:1260. doi: 10.1038/s41467-017-01148-5 (PMC5662612; doi:10.1038/s41467-017-01148-5)
Supplement: Supplementary file 1 — Supplementary Information [file 41467_2017_1148_MOESM1_ESM.pdf]

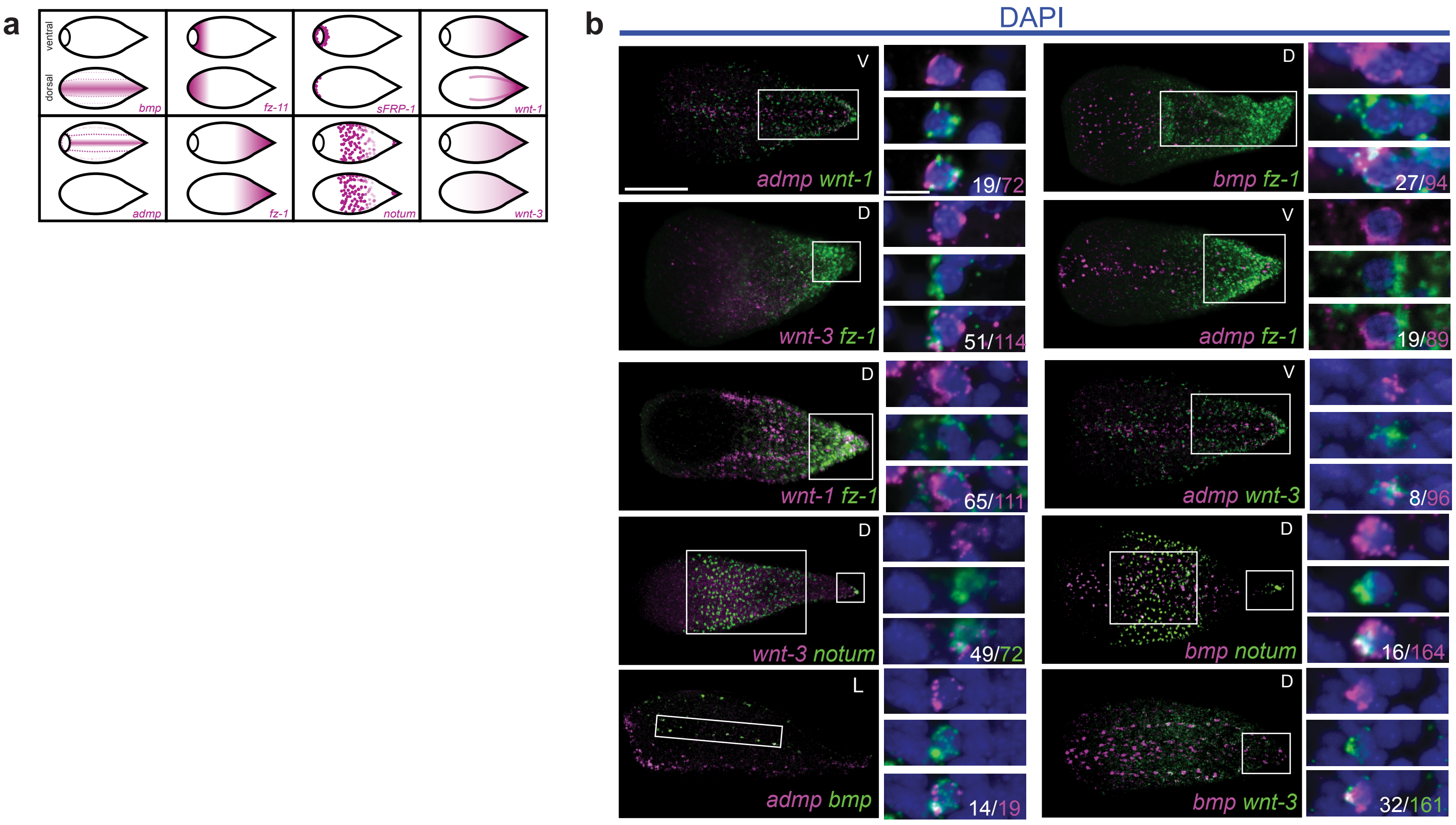

Supplementary Figure 1. Characterization of PCG expression patterns. Related to Figure 1.
a, Wnt and Bmp pathway components are expressed regionally in *Hofstenia*. *bmp* is expressed dorsally, whereas *admp* is expressed ventrally. *sFRP-1* is expressed in the extreme anterior around the mouth. *notum* is expressed throughout the midbody and in the posterior tip. *frizzled-11* is expressed in an anterior gradient. *frizzled-1*, *wnt-1*, and *wnt-3* are all expressed in gradients from the posterior[1]. **b,** Continued from Fig. 1d. 18 RNA probe pairs were used in double-FISH experiments. The denominator for each quantification is the total number of cells in the channel indicated (i.e., magenta or green); the numerator is the total number of double-positive cells for that pair. Some PCG pairs (e.g., *bmp*/*notum*) show infrequent co-expression in the midbody, but are both expressed in muscle. This might indicate that some PCGs are expressed in different subsets of muscle cell types.
Scale bars: **b,** 100 μm, overview image; 10 μm, cell inset.
D, dorsal view. V, ventral view. L, lateral view.
Overview images representative of 5/5 animals examined.

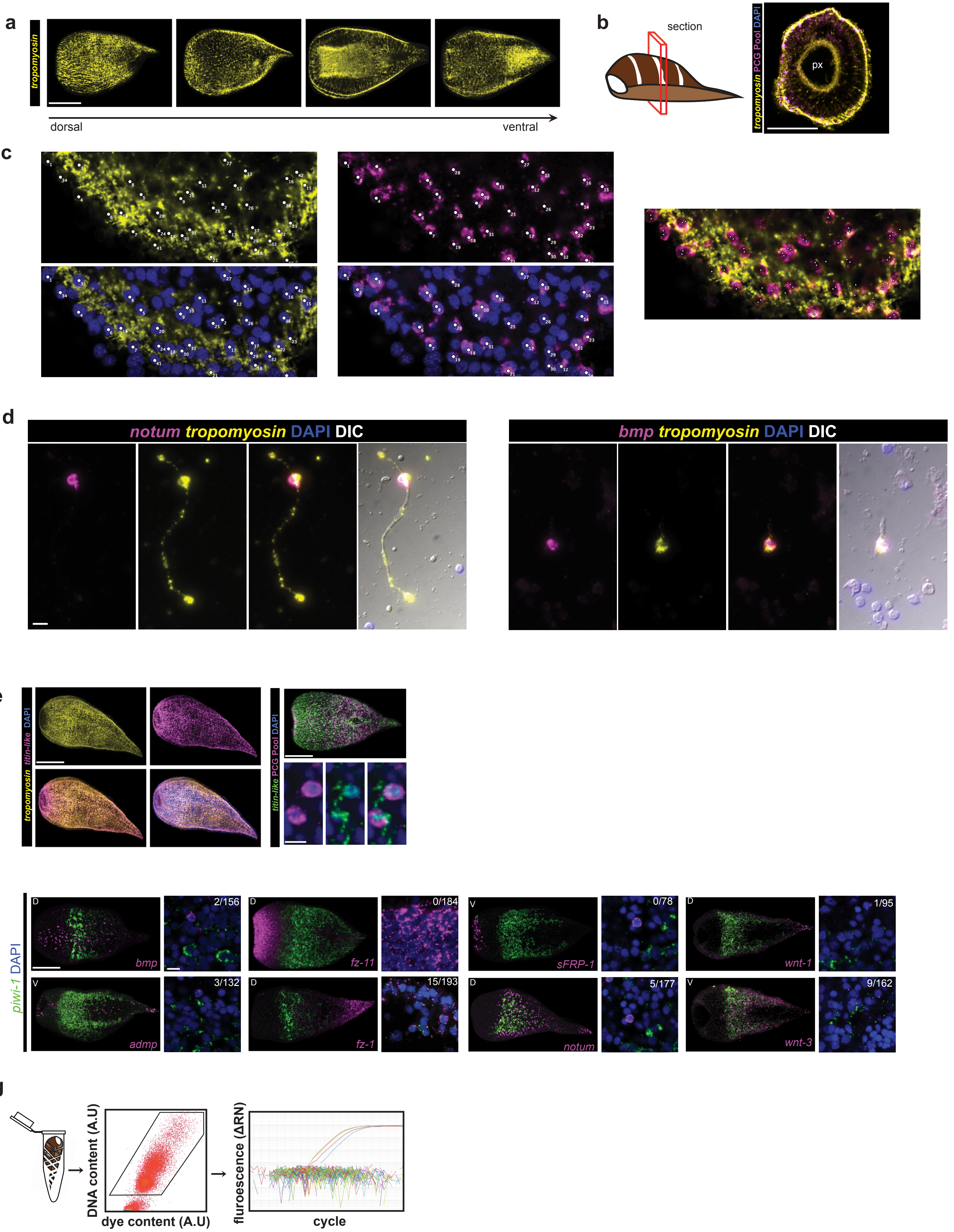

a
tropomyosin
dorsal
ventral
b
section
px
tropomyosin PCG Pool DAPI
c
d
notum tropomyosin DAPI DIC
bmp tropomyosin DAPI DIC
e
tropomyosin titin-like DAPI
titin-like PCG Pool DAPI
f
piwi-1 DAPI
D
bmp
2/156
fz-11
0/184
V
sFRP-1
0/78
wnt-1
1/95
admp
3/132
fz-1
15/193
notum
5/177
wnt-3
9/162
g
DNA content (A.U)
dye content (A.U)
fluroescence (ΔRN)
cycle

Supplementary Figure 2. *Hofstenia* muscle morphology, molecular markers, and co-expression with PCGs. Related to Figure 2.

a, The outermost layer of *Hofstenia* muscle is comprised of longitudinal fibers that run along the anteroposterior axis of the animal. Interior to those fibers are circular fibers that run along the medial-lateral axis and encircle the width of the animal. There are also fibers that point interiorly from the body wall to the pharynx, and the pharynx itself is highly muscularized. All identified morphological muscle subtypes expressed *tropomyosin*. **b,** PCGs were expressed in a common cell layer, directly interior to *tropomyosin*+ muscle fibers. The PCG RNA probe pool was the same as in Fig. 1c, 2b, and 3b. **c,** Example of quantification strategy. First, cells positive for the first channel were marked using expression of channel 1 and DAPI, while blinded to expression in the second channel. Then, cells positive for the second channel were marked using expression of channel 2 and DAPI while blinded to expression in the first channel. Finally, channels were merged to determine co-localization (i.e., cells that were marked in both quantification rounds). **d,** FISH was performed on macerated *Hofstenia* cells. PCG expression was specific to muscle cells (*tropomyosin*+). Observed muscle fibers had only one associated nucleus, consistent with a view that *Hofstenia* muscle cells are mononucleated. **e,** *titin-like* was expressed in *Hofstenia* muscle fibers in a similar pattern to tropomyosin. Co-expression assay with *titin-like* is consistent with data from tropomyosin. The PCG RNA probe pool was the same as Fig. 1c, 2b, 3b, and Supplementary Fig. 2b. **f,** PCGs were not significantly co-expressed with the cycling cell marker *piwi-1*. **g,** Related to Fig. 2c. Animals were macerated into a single-cell suspension. Next, fluorescence activated cell sorting (FACS) was used to isolate all live, nucleated cells using Hoechst and propidium iodine labeling. These cells were sorted singly into individual wells of a 96-well plate. cDNA libraries were amplified and screened by qRT-PCR.

Scale bars: **a,** 100 µm; **b,** 100 µm; **d,** 10 µm, cell inset; **e, f,** 100 µm, overview image; 10 µm, cell inset.

D, dorsal view. V, ventral view. L, lateral view. S, coronal section.

Overview images representative of 5/5 animals examined.

.

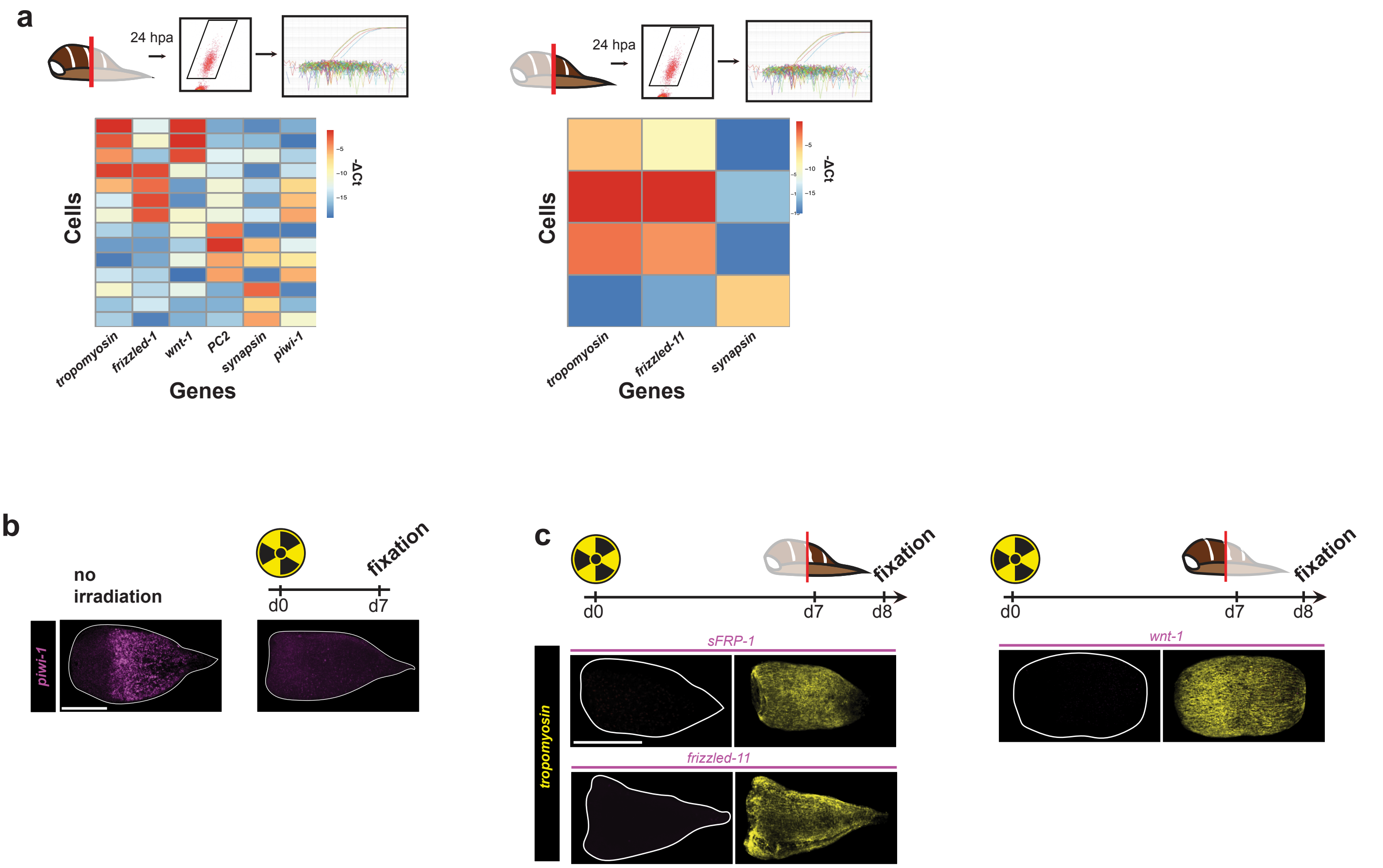

Supplementary Figure 3. Muscle cells express PCGs during regeneration. Related to Figure 3.
a, Single cells from amputated animals were isolated by FACS. The resulting cDNA was screened by qRT-PCR using probes specific to *GAPDH, tropomyosin, frizzled-1, wnt-1, PC2, synapsin,* and *piwi-1* in head fragments undergoing posterior regeneration, and by probes specific to *GAPDH, tropomyosin, frizzled-11, sFRP-1, PC2,* and *synapsin* in tail fragments undergoing anterior regeneration. Any cell without expression of GAPDH was excluded from analysis. Shown are all remaining cells positive for at least one PCG and/or at least one neural marker. No *sFRP-1*(+) or *PC2*(+) cells were found in regenerating tail fragments in this analysis. -ΔCt = -[Ct(gene of interest) - Ct(GAPDH)]. All Ct values greater than 32 or undetected were analyzed as Ct=32. 8/10 PCG-positive cells also expressed *tropomyosin*. 0/10 PCG-positive cells expressed neural markers *PC2* or *synapsin.* **b,** Animals were lethally irradiated with 10,000 rads and assayed for successful ablation of *piwi-1* expression 7 days post-irradiation. **c,** Animals from the same cohort as Fig. 5b and Supplementary Fig. 3b were irradiated and amputated after ablation of *piwi-1* expression (7 days post-irradiation). PCG expression was assayed 24 hpa. The PCGs *sFRP-1*, *frizzled-11,* and *wnt-1* did not show re-expression after amputation in irradiation conditions, suggesting re-expression of these genes requires production of new tissue.

Scale bars: **b, c,** 100 μm.
Overview images representative of 5/5 animals examined.

Primer sets (5'-3') used for qRT-PCR

Gene	Forward primer	Reverse primer
admp	TGGGAAGCACGGTAAAGGAT	AAATCTGATCCTGCCGCAAT
bmp	CCCGTCCTTATGACCACGTT	TTGAACATGCCCAATCTCCA
frizzled-1	TGGAGCAGTAGCAGCTTTGG	TGGTGCGATTGTGAACATGA
frizzled-11	CGCATGAATTGCGATCACTT	GTTTTGGCTTTGTGGGCTTC
notum	GATGGAACTCCAGCGACGTA	GTTTGCACGTTTGTGGCATT
PC2	ACAGTGCAAGTTGGGGTCCT	CAGACCGTTTCTTCCCGAGT
piwi-1	AACGAGGTGGTGGTGTAGCC	GAGCATCAGTGCGACCTCTG
sFRP-1	TGCGAAAGCGTTAAGTCAGC	GGCACTTCTTCGTCCAAAGG
synapsin	GTAATCACCAACCGGCCAAT	GCTGGGAACGGAAGTTTGTC
tropomyosin	CACCAAGCTCGAACAACAGC	GTTGCCTCCGGTGATTTCAG
wnt-1	GCACTGCAGAACGTGGTATTG	TTGACATTCGGAAGCAATGG
wnt-3	TGGTGTTCCTGGATCCTTCC	GGAGCACGAGCCAGTAGAAAG

Supplementary Table 1

Known PCGs in non-planarian organisms

Gene	Organism	Expression pattern	Regenerative Phenotype	Planarian homolog?	Non-planarian reference	Planarian reference
nodal	*Hydra*	Presumptive budding zone foci	Inhibition of Tgf-β pathway leads to radial buds	Unknown	2	N/A
bmp5	*Hydra*	Oral end	Inhibition of Tgf-β pathway leads to radial buds	Yes	2	3, 4, 5
activin1	*Hydra*	Midbody	Inhibition of Tgf-β pathway leads to radial buds	Yes	2	6, 7
activin3	*Hydra*	Midbody	Inhibition of Tgf-β pathway leads to radial buds	Yes	2	6, 7
wnt3	*Hydra*	Oral tip	Overexpression leads to ectopic oral ends	Yes	8	9, 10, 11, 12
wntA	*Nematostella*	Oral end	Wnt pathway overexpression leads to ectopic oral ends	Yes	13, 14	15
wnt1	*Nematostella*	Oral tip	Wnt pathway overexpression leads to ectopic oral ends	Yes	13, 14	9, 10, 12
wnt7	*Nematostella*	Oral tip	Wnt pathway overexpression leads to ectopic oral ends	Yes	13, 14	9, 10, 11, 12
wnt5	*Nematostella*	Oral end	Wnt pathway overexpression leads to ectopic oral ends	Yes	13, 14	11, 12
notum	*Hofstenia*	Midbody, posterior tip	Knockdown leads to ectopic posterior	Yes	1	16
wnt-1	*Hofstenia*	Posterior gradient	Knockdown leads to ectopic anterior	Yes	1	10
admp	*Hofstenia*	Ventral stripes	Knockdown leads to ectopic ventralization	Yes	1	17, 18
bmp	*Hofstenia*	Dorsal stripes	Knockdown leads to ectopic ventralization	Yes	1	3
frizzled-11	*Hofstenia*	Anterior gradient	Inhibition of Wnt pathway leads to ectopic anterior	Yes	1	9, 19, 20, 21
frizzled-1	*Hofstenia*	Posterior gradient	Inhibition of Wnt pathway leads to ectopic anterior	Yes	1	9, 19, 20, 21
sFRP-1	*Hofstenia*	Sharp ring in anterior	Inhibition of Wnt antagonists leads to ectopic posterior	Yes	1	9, 19
wnt-3	*Hofstenia*	Posterior gradient	Inhibition of Wnt pathway leads to ectopic anterior	Yes	1	9, 10, 11, 12

Supplementary Table 2

Supplementary References

1. Srivastava, M., Mazza-Curll, K. L., Van Wolfswinkel, J. C. & Reddien, P. W. Whole-body acoel regeneration is controlled by Wnt and Bmp-Admp signaling. *Curr. Biol.* **24,** 1107–1113 (2014).

2. Watanabe, H. *et al.* Nodal signalling determines biradial asymmetry in *Hydra*. *Nature* **515,** 112-114 (2014)

3. Reddien, P. W., Bermange, A. L., Kicza, A. M., Sánchez Alvarado, A. BMP signaling regulates the dorsal planarian midline and is needed for asymmetric regeneration. *Development* **134,** 4043–4051 (2007).

4. Molina, M. D., Saló, E. & Cebrià, F. The BMP pathway is essential for re-specification and maintenance of the dorsoventral axis in regenerating and intact planarians. *Dev. Biol.* **311,** 79–94 (2007).

5. Orii, H. & Watanabe, K. Bone morphogenetic protein is required for dorso-ventral patterning in the planarian *Dugesia japonica*. *Dev. Growth Differ.* **49,** 345–349 (2007).

6. Gaviño, M. A., Wenemoser, D., Wang, I. E. & Reddien, P. W. Tissue absence initiates regeneration through Follistatin-mediated inhibition of Activin signaling. *Elife* **2,** e00247 (2013).

7. Roberts-Galbraith, R. H. & Newmark, P. A. Follistatin antagonizes Activin signaling and acts with Notum to direct planarian head regeneration. *Proc. Natl. Acad. Sci. U. S. A.* **110,** 1363–1368 (2013).

8. Lengfeld, T. *et al.* Multiple Wnts are involved in *Hydra* organizer formation and regeneration. *Dev. Biol.* **330,** 186–99 (2009).

9. Petersen, C. P. & Reddien, P. W. *Smed-βcatenin-1* is required for anteroposterior blastema polarity in planarian regeneration. *Science* **319,** 327–30 (2008).

10. Petersen, C. P. & Reddien, P. W. A wound-induced Wnt expression program controls planarian regeneration polarity. *Proc. Natl. Acad. Sci. U. S. A.* **106,** 17061–17066 (2009).

11. Gurley, K. A. *et al.* Expression of secreted Wnt pathway components reveals unexpected complexity of the planarian amputation response. *Dev. Biol.* **347,** 24–39 (2010).

12. Adell, T., Salò, E., Boutros, M. & Bartscherer, K. *Smed-Evi/Wntles*s is required for β-catenin-dependent and -independent processes during planarian regeneration. *Development* **136,** 905–910 (2009).

13. Trevino, M., Stefanik, D. J., Rodriguez, R., Harmon, S. & Burton, P. M. Induction of canonical Wnt signaling by alsterpaullone is sufficient for oral tissue fate during

regeneration and embryogenesis in *Nematostella vectensis*. *Dev. Dyn.* **240,** 2673–2679 (2011).

14. Kusserow, A. *et al.* Unexpected complexity of the Wnt gene family in a sea anemone. *Nature* **433,** 156–160 (2005).

15. Kobayashi, C., Saito, Y., Ogawa, K. & Agata, K. Wnt signaling is required for antero-posterior patterning of the planarian brain. *Dev. Biol.* **306,** 714–724 (2007).

16. Petersen, C. P. & Reddien, P. W. Polarized *notum* activation at wounds inhibits Wnt function to promote planarian head regeneration. *Science* **332,** 852–855 (2011).

17. Gaviño, M. A. & Reddien, P. W. A Bmp/Admp regulatory circuit controls maintenance and regeneration of dorsal-ventral polarity in planarians. *Curr. Biol.* **21,** 294–9 (2011).

18. Molina, M. D. *et al.* Noggin and noggin-like genes control dorsoventral axis regeneration in planarians. *Curr. Biol.* **21,** 300–305 (2011).

19. Gurley, K. A., Rink, J. C. & Sánchez Alvarado, A. Beta-catenin defines head versus tail identity during planarian regeneration and homeostasis. *Science* **319,** 323–7 (2008).

20. Lander, R. & Petersen, C. P. Wnt, Ptk7, and FGFRL expression gradients control trunk positional identity in planarian regeneration. *eLife* **5,** 1–19 (2016).

21. Scimone, M. L., Cote, L. E., Rogers, T. & Reddien, P. W. Two FGFRL-Wnt circuits organize the planarian anteroposterior axis. *eLife* **5,** e12845 (2016).
