# Supplementary material for: Acoel regeneration mechanisms indicate an ancient role for muscle in regenerative patterning
Source: Nat Commun. 2017 Oct 30;8:1260. doi: 10.1038/s41467-017-01148-5 (PMC5662612; doi:10.1038/s41467-017-01148-5)
Supplement: Supplementary file 2 — Peer Review File [file 41467_2017_1148_MOESM2_ESM.pdf]

Editorial Note: this manuscript has been previously reviewed at another journal that is not operating a transparent peer review scheme. This document only contains reviewer comments and rebuttal letters for versions considered at Nature Communications.
